# Optimising preclinical mouse models: lesson from Batten disease

**Published:** 2015-04

Juvenile Batten disease (also known as neuronal ceroid lipofuscinosis) is a neurodegenerative disorder that affects children between 4 and 10 years of age, and for which there are no treatments. It is characterised by the intracellular accumulation of lipopigments and is most commonly caused by mutations in the ceroid lipofuscinosis neuronal 3 (*CLN3*) gene, which encodes a transmembrane protein expressed in lysosomes and endosomes. Preclinical animal models are important to advance knowledge of disease pathophysiology and to identify therapeutic targets. However, some factors, including gender and genetic background, can hinder the expression of the appropriate disease phenotype. David Pearce’s group sought to identify the most suitable mouse model for juvenile Batten disease. They used *Cln3*-knockout (*Cln3*^−/−^) and *Cln3^Δex7/8^*-knock-in mice, two common models of this disease, on different genetic backgrounds (129S6/SvEv and C57BL/6J). They evaluated female and male mice from both models at different months of age for several neurological alterations that can be used as readouts in therapeutic studies. They found that *Cln3*^−/−^ male mice on the 129S6/SvEv background better resemble the neurological impairments associated with the human condition. The study shows that the genetic background and gender can highly influence the disease phenotype, effects that should be carefully taken into account to choose appropriate preclinical models of disease. **Page 351**

